# PB.45: Evaluation of a pilot MRI breast surveillance project for young women at high risk of breast cancer

**DOI:** 10.1186/bcr3545

**Published:** 2013-11-08

**Authors:** S De Souza, N Evans, R Fox, A Murray

**Affiliations:** 1 Screening Division, Public Health Wales, Cardiff, UK; 2 All Wales Medical Genetics Service, Cardiff, UK

## Introduction

NICE Clinical Guidelines recommend that young women at high risk of breast cancer are offered annual surveillance with MRI and digital mammography. A feasibility study was run over 3 years (2010 to 2013) for women with known *BRCA1 *or *BRCA2 *mutations and those with, or at high risk of, a *TP53 *gene mutation. We aimed to explore the practicalities of delivering a surveillance service for these high-risk women.

## Method

Eligible women were identified by the All-Wales Medical Genetics Service and offered annual MRI, with digital mammography where appropriate, at three centres across Wales. Administration was managed by Breast Test Wales.

## Results

Qualitative feedback showed that women were willing to travel for appointments and the service was acceptable. Only 12 of the 38 participants attended all years. This is a dynamic group - some develop cancer, others opt for risk-reducing surgery, and each year some were ineligible as they were pregnant or breastfeeding. Administration of the system was complex, involving different professional groups working across sites. Standards for waiting times for results were not met. Few radiologists in Wales meet the English standard of 100 breast MRI examinations per year to be eligible as readers. This poses a challenge for service delivery. MRI scanner capacity is also limited.

## Conclusion

The enthusiasm of the multidisciplinary team ensured the project was successful and the service delivered. However, there were major administrative and resource challenges, and the pilot service model was unsustainable. The evaluation will be used to inform decisions on future service provision.

